# Magnetic Helicity and the Solar Dynamo

**DOI:** 10.3390/e21080811

**Published:** 2019-08-19

**Authors:** John V. Shebalin

**Affiliations:** Space Weather Laboratory, Department of Physics and Astronomy, George Mason University, Fairfax, VA 22030, USA; jshebali@gmu.edu

**Keywords:** solar dynamo, magnetohydrodynamics, thermodynamics, turbulence

## Abstract

Solar magnetism is believed to originate through dynamo action in the tachocline. Statistical mechanics, in turn, tells us that dynamo action is an inherent property of magnetohydrodynamic (MHD) turbulence, depending essentially on magnetic helicity. Here, we model the tachocline as a rotating, thin spherical shell containing MHD turbulence. Using this model, we find an expression for the entropy and from this develop the thermodynamics of MHD turbulence. This allows us to introduce the macroscopic parameters that affect magnetic self-organization and dynamo action, parameters that include magnetic helicity, as well as tachocline thickness and turbulent energy.

## 1. Introduction

The interior of the Sun consists of three major parts: the energy-producing core (0–0.25 $R_{⊙}$), the stably-rotating radiative zone (0.25–0.7 $R_{⊙}$), and the turbulent convection zone (0.7–1.0 $R_{⊙}$) [1]. Separating the radiative and the convective zones is a turbulent transition layer called the tachocline [2], of a thickness of 0.02–0.04 $R_{⊙}$ [3], which may be oscillating [4]. The solar dynamo is believed to reside in the tachocline [5,6], although further shaping and self-organization of the magnetic field occur in the convection zone [7,8].

Here, we focus on the tachocline, review the equilibrium statistical mechanics of ideal magnetohydrodynamic (MHD) turbulence [9], and use this to introduce a thermodynamics of MHD turbulence applicable to the solar tachocline. Compressible MHD, of course, must be used to understand the whole Sun, (e.g., [10]) used an anelastic spherical harmonics code that models the radiation and convective layers, while also producing a tachocline of thickness 0.04 $R_{⊙}$, commensurate with [2]; we note that fully-compressible codes are also available [11,12]. However, since the tachocline is a layer thin enough to be treated, for our purposes, as incompressible, the statistical analysis of [9] can be applied to this case. Although a tachocline does not seem necessary in all stars that display global magnetic activity [8], the solar tachocline provides the basic structure for our analysis.

Thus, we assume the tachocline is a thin rotating spherical shell containing incompressible MHD turbulence and model it as having homogeneous boundary conditions. This approach is similar to one that had no inner boundary, which [13] and [14] studied computationally at very low resolution. The model system of [9] used a Galerkin expansion with basis functions consisting of a combination of spherical Bessel and Neumann functions of radius, along with vector spherical harmonic cofactors representing angular (colatitude and azimuth) variation. This is a “spectral method” that is useful for both theoretical analysis and numerical simulation (although a fully-implemented spectral transform method code still awaits development).

That the statistical mechanics of ideal MHD turbulence is applicable to real systems in quasi-equilibrium was shown by [15,16]. It should also be applicable to stars such as the Sun because the kinetic and magnetic Reynolds numbers are so high [17]. Dynamical modes with lower wavenumbers (corresponding to larger length-scales) have negligible dissipation, while large dissipation wavenumbers ensure that there are many larger-scale modes with close to ideal behavior. Energy loss does occur, and equilibrium requires, of course, that energy must flow into the tachocline, an energy flow provided by hydrogen fusion in the solar core. In addition, the product of magnetic helicity and the smallest wavenumber in the spherical shell model must be relatively large to create a predominantly dipole magnetic field. Magnetic helicity may be created by differential rotation [18], perhaps at the interface between the radiation zone and the tachocline [19]. The other cofactor, the fundamental wave number, that influences dynamo action varies as the inverse of the thickness of the turbulent spherical shell; thinner shells mean larger wavenumbers, though if too thin, increased dissipation. These two parameters (magnetic helicity and shell thickness), as well as others, will be considered presently with regard to how their variability affects dynamo action in the tachocline.

## 2. Spherical Shell Models

The Sun, like the Earth, contains a turbulent magnetofluid where dynamo action occurs. For the Earth, this magnetofluid can be modeled as being incompressible and contained in a spherical shell with homogeneous boundary conditions (b.c.s) [9]. Homogeneous b.c.s reflect the entrainment of the magnetic field by fluid velocity and connect the inner magnetic field to the exterior potential field through the continuity of transverse poloidal components. In this model, spherical coordinates (r,θ,ϕ) are used with the radius *r* measured in terms of the inner radius of the tachocline, $R_{I}=0.7R_{⊙}$, so that r=1 is the inner boundary, $r=r_{o}=1+h$ is the outer boundary and *h* is the nondimensional thickness, where 0.03≤h≤0.06 is an approximate range for the tachocline. The velocity and magnetic fields are expanded in terms of spherical Bessel and Neumann functions coupled with vector spherical harmonics; as an example, the magnetic field is, in terms of toroidal coefficients $b_{lmn}$ and poloidal coefficients $a_{lmn}$,

(1) $$b\left(x,t\right)=\sum_{l,m,n}\left[b_{lmn}\left(t\right)T_{lmn}\left(x\right)\left(r,\theta ,\phi \right)+a_{lmn}\left(t\right)P_{lmn}\left(x\right)\left(r,\theta ,\phi \right)\right].$$

The summation indices in (1) have the ranges 1≤l≤L, −l≤m≤l, and 1≤n≤N. When expansion (1) and a similar expansion (with toroidal $v_{lmn}$ and poloidal $w_{lmn}$ coefficients) for the velocity field are put into the MHD equations, the result is a dynamical system whose phase space is defined by the set of all coefficients. The total number of combination of indices l,m,n is K=NL(L+2), so the phase space dimension is M=4K.

In (1), the poloidal basis functions are $P_{lmn}\left(x\right)=\nabla \times T_{lmn}\left(x\right)$, and the toroidal basis functions are:

(2) $$T_{lmn}\left(x\right)\left(r,\theta ,\phi \right)=F_{ln}g_{l}\left(k_{ln}r\right)r\times \nabla Y_{lm}\left(\theta ,\phi \right).$$

$F_{ln}$ is a normalizing constant, $Y_{lm}\left(\theta ,\phi \right)$ a spherical harmonic, and $g_{l}\left(k_{ln}r\right)$, $1\leq r\leq r_{o}$, is:

(3) $$g_{l}\left(k_{ln}r\right)=n_{l}\left(k_{ln}r_{o}\right)j_{l}\left(k_{ln}r\right)-j_{l}\left(k_{ln}r_{o}\right)n_{l}\left(k_{ln}r\right).$$

Here, $j_{l}\left(z\right)$ and $n_{l}\left(z\right)$ are spherical Bessel and Neumann functions, respectively. Since $g_{l}\left(k_{ln}r_{o}\right)\equiv 0$, homogeneous b.c.s are fully satisfied if $g_{l}\left(k_{ln}\right)=0$, which gives us the wavenumbers $k_{ln}$. When the wavenumbers become large, asymptotic forms of $j_{l}\left(z\right)$ and $n_{l}\left(z\right)$ given by [20] tell us that:

(4) $$\lim_{k_{ln}\to \infty }g_{l}\left(k_{ln}r\right)=\frac{1}{k_{ln}^{2}r_{o}r}\sin\left[k_{ln}\left(r_{o}-r\right)\right].$$

Thus, for large wavenumbers,

(5) $$k_{ln}≅n\pi /h,\;h=r_{o}-1,\;n=1,2,\cdots .$$

**Figure 1:**
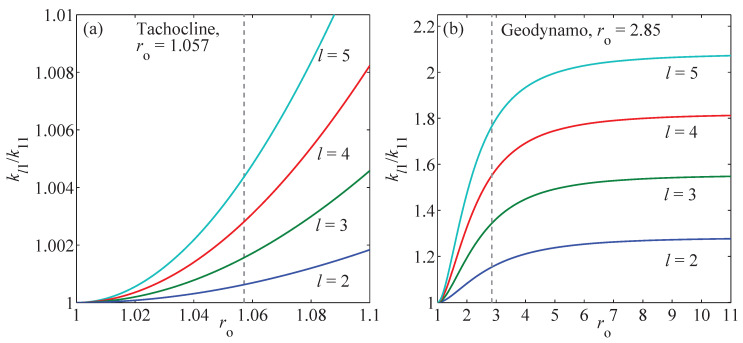
Ratio of wave vectors $k_{l1}/k_{11}$, l=2,⋯,5, with respect to $r_{o}$. (**a**) For the thin tachocline, $r_{o}=1.057$. (**b**) For the Earth’s outer core geodynamo, $r_{o}=2.85$. Notice that $k_{l1}<k_{l+1,1}$ for $r_{o}>1$.

In other words, wavenumbers tend to become large and seemingly independent of *l* as $r_{o}\to 1$ or as n→∞. For a tachocline of thickness 0.04 $R_{⊙}$, h=0.057, and the exact $k_{11}≅55.133$, while π/h≅55.116; for the Earth’s outer core, h=1.85 and the exact $k_{11}≅1.864$, while π/h≅1.698. However, as Figure 1 shows, the independence of $k_{ln}$ from *l* is not achieved until $r_{o}=1$, and at that limit, the flow is not 2D, but is, in fact, no longer representable by the expansion because all of the $g_{l}\left(k_{ln}r\right)\to \sin\left(n\pi \right)=0$. The closest one can come to a 2D spherical model is to use, for example, only the wave numbers $k_{l1}$, or perhaps $k_{ll}$, or some similar reduction. This preserves the essential relationship $P_{lmn}\left(x\right)=\nabla \times T_{lmn}\left(x\right)$, while reducing the phase space dimension from NL(L+2) to L(L+2). This is “as simple as possible, but no simpler.”

The Sun, of course, is compressible, so that an incompressible model is perhaps a better approximation for thinner spherical shells rather than thicker. Nevertheless, turbulence is due to nonlinearity with compressibility being important for motions that generate and are affected by sound waves. In considering the tachocline as the seat of the solar dynamo and long-term magnetic variability, we may expect that compressibility plays a secondary role compared to the dynamics of incompressible MHD turbulence. In addition, an incompressible model allows us to apply statistical mechanics to ideal MHD turbulence in a straightforward manner. This application produces some novel results concerning the solar dynamo, and it is to these that we now turn.

## 3. Statistical Mechanics of MHD Turbulence

The statistical mechanics of ideal, rotating MHD turbulence is based on a probability density function of the form:

(6) $$D=Z^{-1}\exp\left(-\alpha E-\gamma H_{M}\right),.$$

This probability density function for rotating MHD turbulence depends on energy *E* (where $E=E_{K}+E_{M}$) and magnetic helicity $H_{M}$ because these are ideal invariants [21]. Expressions for kinetic energy $E_{K}$, magnetic energy $E_{M}$, and magnetic helicity $H_{M}$ are given in [9]. Here, we will write these in terms of helical variables:

(7) $$b_{lmn}^{\pm }=\frac{1}{\sqrt{2}}\left[b_{lmn}\pm k_{ln}a_{lmn}\right],\;w_{lmn}^{\pm }=\frac{1}{\sqrt{2}}\left[w_{lmn}\pm k_{ln}v_{lmn}\right].$$

The $b_{lmn}^{\pm }$ have ± magnetic helicity, and the $w_{lmn}^{\pm }$ have ± kinetic helicity. Using these, $E_{K}$, $E_{M}$, and $H_{M}$ are:

(8) $$\begin{array}{ccc} E_{K} & = & \frac{1}{2}\sum_{l,m,n}\left(|w_{lmn}^{+}{|}^{2}+{\left|w_{lmn}^{-}\right|}^{2}\right), \end{array}$$

(9) $$\begin{array}{ccc} E_{M} & = & \frac{1}{2}\sum_{l,m,n}\left(|b_{lmn}^{+}{|}^{2}+{\left|b_{lmn}^{-}\right|}^{2}\right), \end{array}$$

(10) $$\begin{array}{ccc} H_{M} & = & \frac{1}{2}\sum_{l,m,n}\left(|b_{lmn}^{+}{|}^{2}-{\left|b_{lmn}^{-}\right|}^{2}\right). \end{array}$$

Again, the total number of terms in these summations is K=NL(L+2), and the dimension of the associated phase space is M=4K (*K* represents the effective number of dynamically-active modes with wavenumbers less than the dissipation wavenumber of the turbulent MHD dynamo layer in the Sun). Further details concerning the statistical mechanics of ideal MHD turbulence can be found elsewhere [9,22]. Here, we will examine a few features that are pertinent to solar dynamism.

If the expressions for *E* and $H_{M}$ are placed into (6), it is straightforward to determine the partition function and from this the expectation values of the means and variances for the variables $b_{lmn}^{\pm }$ and $w_{lmn}^{\pm }$. The means are expected to be zero, but in numerical simulations of both ideal and real MHD turbulence in a periodic box, it has been found that the dynamical time-averages of the magnetic field coefficients with the smallest wavenumber can be very large compared to their standard deviations [15,16,22,23,24,25]. This is an example of broken ergodicity [26]. Since the statistical mechanics of ideal MHD turbulence is essentially the same for the spherical shell [9] as for the periodic box [22], numerical simulations should yield similar results in the spherical case, once the necessary spectral transform method codes are developed (this is a “computational grand challenge” for those who wish to attempt it).

The expectation values of energy and magnetic helicity must match their initial values E and $H_{M}$ when these are conserved, i.e., $\langle E\rangle \equiv E$ and $|\langle H_{M}\rangle |\equiv H_{M}$. This, in turn, tells us that α and γ in (6) can be expressed as:

(11) $$\alpha =\frac{2K}{E-\varphi },\;\gamma =-\frac{2\varphi -E}{H_{M}}\alpha .$$

Again, K=NL(L+2) is the number of modes. The quantity $\varphi =\langle E_{M}\rangle$ is the expectation value of the magnetic energy, which is not an ideal invariant like *E* and $H_{M}$. Initially, φ is unknown, but can be determined by minimizing the entropy functional $\sigma \left(\varphi \right)=-\langle \ln D\rangle$. The result of doing so leads to the minimizing value $\varphi =\varphi_{o}$, where:

(12) $$\varphi_{o}≅\frac{1}{2}\left(E+k_{11}H_{M}\right)-\frac{1}{4}\epsilon \left(E-k_{11}H_{M}\right).$$

Here, ϵ=m/2K where *m* is the number of modes that have wavenumbers $k_{ln}$ equal to the smallest wavenumber $k_{11}$.

The expected energies of the dynamical variables with respect to helicity are:

(13) $$\begin{array}{ccc} \frac{1}{2}\langle |w_{lmn}^{\pm }{|}^{2}\rangle & = & \frac{E-k_{11}H_{M}}{4K},\;k_{ln}\geq k_{11}, \end{array}$$

(14) $$\begin{array}{ccc} \frac{1}{2}\langle |b_{lmn}^{-}{|}^{2}\rangle & = & \frac{k_{ln}}{k_{ln}+k_{11}}\frac{E-k_{11}H_{M}}{4K},\;k_{ln}\geq k_{11}, \end{array}$$

(15) $$\begin{array}{ccc} \frac{1}{2}\langle |b_{lmn}^{+}{|}^{2}\rangle & = & \frac{k_{ln}}{k_{ln}-k_{11}}\frac{E-k_{11}H_{M}}{4K},\;k_{ln}>k_{11}, \end{array}$$

(16) $$\begin{array}{ccc} \frac{1}{2}\langle |b_{1m1}^{+}{|}^{2}\rangle & = & \frac{k_{11}H_{M}}{m},\;k_{ln}=k_{11}. \end{array}$$

The sum of these over independent modes $k_{ln}$ is E plus a term of O($K^{-1}$), as it should be. For a spherically-symmetric model, the three variables $b_{1m1}^{+}$m=−1,0,+1 of (16) are real and supply the three components of the quasi-steady magnetic dipole moment vector. The sum of their energies energies is $E_{d}$, the energy of the magnetic dipole field, while the remainder with respect to E is the turbulent energy $E_{turb}$:

(17) $$E_{d}=k_{11}H_{M},\;E_{turb}=E-E_{d}.$$

The turbulent energy $E_{turb}$ has essentially equal kinetic and magnetic parts, as can be seen by summing the two terms given in (13) and comparing this with the sum of (14) and (15). Clearly, the dipole energy $E_{d}$ becomes large as the product $k_{11}H_{M}$ becomes large.

If one considers the dynamical equations for the variables $w_{lmn}^{\pm }$ and $b_{lmn}^{\pm }$, it is found the their mean square fluctuations are of order $K^{-1}$[9]. This is the same order of magnitude as their expected variances (13)–(15). The result of this is that the dynamical mean values of $w_{lmn}^{\pm }$ and $b_{lmn}^{\pm }$ for all except $b_{1m1}^{+}$ match their expected values, i.e., they are zero-mean random variables. In the case of $b_{1m1}^{+}$, the mean square fluctuations are also of order $K^{-1}$, but the expectation value (16) is of order one. Thus, the three components $b_{1m1}^{+}$, m=−1,0,+1, define a dipole moment vector that has constant magnitude and direction up to order $K^{-1/2}$, and so, it does not match its expectation value of zero: we have the phenomenon of broken ergodicity [26]. Notice that the dipole part plays a preeminent role and that the quadrupole and higher order modes are “just part of the noise”.

In periodic box models, this dipole moment vector tends to align itself with a rotation axis, if one is present in ideal MHD turbulence [22]. However, in dissipative, driven MHD turbulence, this alignment can be affected by the manner in which the system is forced [15,16]. If the angular rotation vector is in the *z*-direction, alignment in the spherical shell model occurs because the component $b_{101}^{+}$ becomes large dynamically, while the components $b_{1,\pm 1,1}^{+}$ become much smaller, i.e., we have broken symmetry. When $b_{101}^{+}$ is large, the toroidal and poloidal parts have equal energy; when either the toroidal or poloidal part is negligible, the dipole component of the magnetic field is of the same size as the other multipole components, i.e., the dynamo has shut off. Thus, the purely-toroidal to purely-poloidal cycle of mean-field dynamo theory [4] does not appear to be a viable process (further comments on the non-viability of mean-field dynamos will be given in Section 5).

## 4. Thermodynamics of MHD Turbulence

Using the previous results, the entropy $S=\sigma (\varphi_{o})$ can be written as:

(18) $$S=M\ln\left[\pi e\left(\frac{E_{turb}}{M}\right)\right].$$

In the above expression, the total number of interacting variables is M=4K, and again, $E_{turb}=E-E_{d}$ is the turbulent energy, where $E_{d}=k_{11}H_{M}$ is the energy of the quasi-stationary dipole magnetic field.

Defining the shell thickness as $h=r_{o}-1$ and using (5) give $k_{11}≅\pi /h$ for a thin shell. Furthermore, the volume of the thin shell is $V=4\pi R_{I}^{2}h$ and fundamental wavenumber is $\kappa \equiv k_{11}$, for brevity. Thus, the fundamental equation for the thermodynamics of MHD turbulence is:

(19) $$S=M\ln\left[\frac{\pi e}{M}\left(E-E_{d}\right)\right],\;E_{d}=\kappa H_{M}.$$

The first law of MHD turbulence thermodynamics is then:

(20) $$dS=\frac{1}{T}dE+\frac{p}{T}dV-\frac{\mu }{T}dM-\frac{\kappa }{T}dH_{M}.$$

The MHD turbulent temperature *T*, pressure *p*, chemical potential μ, and fundamental wavenumber κ are:

(21) $$T=\frac{E_{turb}}{M},\;p=\frac{E_{d}}{V},\;\mu =-T\ln\left(\pi T\right),\;\kappa =\frac{E_{d}}{H_{M}}.$$

The extensive thermodynamic parameters are *S*, E, $V=4\pi R_{I}^{2}h$, and $H_{M}$, while the intensive ones *T*, *p*, κ, and μ.

**Figure 2:**
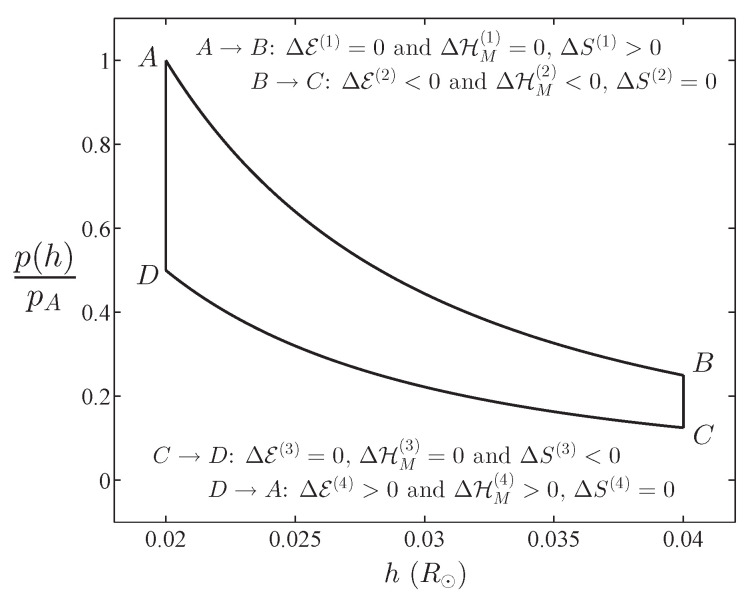
Example of a thermal cycle for a thin tachocline of height *h*, where the volume of the shell is $V=4\pi R_{I}^{2}h$.

Let us consider a thermodynamic cycle in which a thermal engine, using tachocline volume oscillations takes in magnetic helicity and heat from a higher-temperature reservoir, creates a dipole magnetic field, and passes this on to a lower temperature reservoir. In Figure 2, we show a hypothetical thermal cycle; as indicated there, the four parts of the cycle are: (1) A→B, expansion; (2) B→C, constant volume pressure decrease; (3) C→D, compression; and (4) D→A, constant volume pressure increase. During isentropic expansion and compression in this cycle, E and $H_{M}$ are conserved; during (1) expansion, dipole energy $E_{d}$ decreases, while *T* and *S* decrease; during (3) compression, $E_{d}$ increases, while *T* and *S* increase. Of course, reversibility requires that ΔS=0 around the cycle.

This hypothetical model of MHD turbulence in the tachocline is a thermodynamic system that is embedded in the much larger thermodynamic system of the solar interior. Please note that this is a “thermodynamics of MHD turbulence” as opposed to the “magnetothermodynamics” of a plasma treated as a magnetized, ionized gas [27]. If the tachocline is a thermal engine that undergoes a cycle of some sort from one equilibrium state to another and back again, we see that $E_{d}$ oscillates between maximum and minimum values. However, there remain many questions to be answered: How strongly or weakly is the MHD turbulence coupled to rest of the solar interior? What is the mechanism that drives the thermal engine? How does tangential shear inject energy and magnetic helicity into the tachocline? How is the temperature of MHD turbulence related to the temperature of the surrounding plasma? How do the degrees-of-freedom M depend on temperature and volume? Answering these, or attempting to do so, is beyond the scope of the present work. Our hope is that the introductory and novel results presented here will inspire others to move the subject forward.

## 5. Discussion

Here, the statistical mechanics of MHD turbulence in a thin spherical shell was presented, and from these results, the thermodynamics of MHD turbulence was developed. The general statistical result was that MHD turbulence, per se, generates a large, quasi-stationary dipole magnetic field if the product of fundamental wavenumber and absolute value of magnetic helicity is relatively large compared to the turbulent energy. The dynamical system, to reiterate, is the collection of modes that are revealed through the spherical Bessel–Neumann function, vector spherical harmonic representation of the velocity, and magnetic fields. However, once the thermodynamics was formulated, we moved from the very large phase space of coefficients back into the physical system described by a few intensive and a few extensive macroscopic variables.

The development here is based on equilibrium states, but as [28] wrote, “it must be conceded that our primary interest is frequently in processes rather than in states.” Treating processes, such as solar activity, requires perhaps that these results be extended into irreversible thermodynamics [29]. This includes relating the thermodynamics of MHD turbulence to the thermodynamics of the plasma that serves as a host for MHD turbulence. This is a long-term effort, far beyond the scope of the present work, though hopefully a challenge that researchers will take up.

This approach is “physics-based” and, we believe, a door opening into a viable alternative to mean-field electrodynamics (MFE) [30]. MFE assumes that the Reynolds-averaged electromotive force, modeling turbulent action at smaller length scales, is a function of an assumed mean magnetic field; this Reynolds-averaged electromotive force is represented by a series of increasing derivatives of the mean magnetic field (Equation (2.8) of [30]), whereby the magnetic induction equation gains a term linear in this mean magnetic field, guaranteeing its subsequent growth. First, such a linear term appears nowhere in the general Ohm’s law [31]. Second, when long-time mean-squared averages of the nonlinear and dissipative terms in the magnetic induction equation are compared by numerical simulation, they are found to be equal in magnitude, i.e., in equilibrium, they cancel each other on average, as seen in Figure 2 of [15]. In the symmetric case, the MFE coefficients α and β discussed following Equation (2.9) of [30] must be α=0 and β=−η (as opposed to β≫η [30]). Thus, in an equilibrium state, MFE is trivially correct. In nonequilibrium, where α>0 presumably explains the growth of the mean field, it is not needed because it is the inverse cascade and broken ergodicity of MHD turbulence that create the large, quasi-stationary mean magnetic field without any need for an imposed “α-effect”. MHD contains all that is necessary.

## 6. Conclusions

Here, we presented a novel and viable approach to understanding solar dynamism. We assumed that the solar tachocline contains MHD turbulence, which can be treated as incompressible because of the thinness of the tachocline and which satisfies homogeneous boundary conditions. In this model system, the velocity and magnetic fields are expressed in terms of spherical Bessel function, spherical harmonic expansions, allowing a transformation of the partial differential equations of MHD into a very large set of coupled, nonlinear ordinary differential equations where the primary variables are helical, time-dependent expansion coefficients. This dynamical system has two ideal invariant integrals for a rotating spherical shell, energy and magnetic helicity, and on these, an equilibrium statistical mechanics can be based. Developing the statistical mechanics leads to a new and more precise expression for the entropy of the model system and, from this, a novel formulation of the thermodynamics of MHD turbulence. The general importance of these results is that the statistical mechanics of MHD turbulence explains how a dominant, quasi-stationary dipole magnetic field arises, while the associated thermodynamics identifies macroscopic parameters that affect magnetic self-organization and dynamo action.
